# Supplementary material for: Effect of long-chain polyunsaturated fatty acids in infant formula on long-term cognitive function in childhood: A systematic review and meta-analysis of randomised controlled trials
Source: PLoS One. 2020 Nov 5;15(11):e0241800. doi: 10.1371/journal.pone.0241800 (PMC7644261; doi:10.1371/journal.pone.0241800)
Supplement: S1 File — (DOCX) [file pone.0241800.s001.docx]

**S1 Table. Extracted estimates and sources**

| Trial cohort | Outcome | Age (years) | Group | Mean | SD | Total | Source |
| --- | --- | --- | --- | --- | --- | --- | --- |
| Term ENG: 2 centres (1993-95) | WPPSI | 4.5 | Intervention | 103.9 | 14.80 | 90 | Unpublished manuscript provided by UCL (Table 3 and Table 4) |
|  | WPPSI | 4.5 | Control | 109.7 | 12.70 | 94 |  |
|  | WASI | 16 | Intervention | 91.94 | 9.13 | 25 |  |
|  | WASI | 16 | Control | 96.83 | 12.60 | 18 |  |
| Term Europe: 6 centres‎ (1992-93) | WPPSI | 6 | Intervention | 98 | 14.80 | 70 | Willatts et al. (2013), p.540S Table 4 |
|  | WPPSI | 6 | Control | 100.9 | 16.20 | 75 |  |
| Term US: 3 centres‎ ‎(1992-93)‎ | PPVT | 3.3 | Intervention | 96.6 | 15.80 | 34 | Auestad et al. (2003), p.e181 Table 4 |
|  | PPVT | 3.3 | Control | 97.3 | 14.20 | 35 |  |
|  | Stan-Bin | 3.3 | Intervention | 101 | 13.00 | 34 |  |
|  | Stan-Bin | 3.3 | Control | 103 | 15.00 | 35 |  |
| Term NL: Groningen ‎(1997-99) | WASI | 9 | Intervention | 97.3 | 10.71 | 91 | Email Hadders-Algra 2018-01-23, provided table |
| Term NL: Groningen ‎(1997-99) | WASI | 9 | Control | 98.43 | 12.57 | 123 |  |
| Term US: DIAMOND Kansas site (2002-04) | WPPSI | 6 | Intervention | 96.6 | 15.28 | 15 | Email Colombo 2018-01-17, provided table |
|  | WPPSI | 6 | Control | 89.93 | 12.73 | 15 |  |
| Term US: Dallas ‎(1993-95) | WPPSI | 4 | Intervention | 107.5 | 12.78 | 17 | Birch et al. (2007), Table 2, Presented only SEs |
|  | WPPSI | 4 | Control | 101 | 11.33 | 19 |  |
| Term US: DIAMOND Dallas site (2002-04) | BBCS | 2.5 | Intervention | 100.09 | 8.60 | 23 | Drover et al. (2012), p.889 Table 2 |
|  | BBCS | 2.5 | Control | 101.28 | 10.63 | 19 |  |
|  | PPVT | 3.5 | Intervention | 99.35 | 10.88 | 23 | Drover et al. (2012), p.889 Table 3 |
|  | PPVT | 3.5 | Control | 104.89 | 10.57 | 19 |  |
| Preterm ENG: ‎2 centres (1993-96) | WASI | 16 | Intervention | 85.6 | 13.90 | 7 | UCL provided database |
|  | WASI | 16 | Control | 103.2 | 13.60 | 10 |  |
| Preterm SCT: Glasgow ‎(1995-97) | WASI | 10 | Intervention | 93.705 | 11.56 | 50 | UCL provided database |
|  | WASI | 10 | Control | 93.873 | 11.54 | 57 |  |

UCL = University College London

**S2 Table. Risk of bias assessment / data extraction template**

(Questions from Cochrane Risk of Bias Tool)

| Study details | ID | Term US: 3 centres‎ ‎(1992-93)‎ | Term Europe: 6 centres‎ (1992-93) | Term ENG: 2 centres (1993-95) | Term US: Dallas ‎(1993-95) | Term NL: Groningen ‎(1997-99) | Term US: DIAMOND Dallas site (2002-04) | Term US: DIAMOND Kansas site (2002-04) | Preterm ENG: ‎2 centres (1993-96) | Preterm SCT: Glasgow ‎(1995-97) | |
| --- | --- | --- | --- | --- | --- | --- | --- | --- | --- | --- | --- |
|  | Outcome | Stanford Binet | WPPSI | WPPSI | WPPSI | WASI | PPVT | WPPSI | WASI | WASI | |
|  | Result (MD) | -2.00 (-8.62, 4.62) | -2.90 (-7.91, 2.11) | -5.80 (-9.84, -1.76) | 6.5 (-1.43, 14.43) | -1.13 (-4.26, 2.00) | -5.54 (-12.05, 0.97) | 6.67 (-3.38, 16.72) | -17.60 (30.91, 4.29) | -0.17 (-4.67, 4.33) | |
|  | Aim | assignment to intervention | assignment to intervention | assignment to intervention | assignment and completion of intervention | assignment to intervention | assignment to intervention | assignment to intervention | assignment to intervention | assignment to intervention | |
|  | Sources | journal articles, personal communication with the trialists | journal articles, personal communication with the trialists, conference abstracts | journal articles, personal communication with the trialists, trial protocol, original data | journal articles, personal communication with the trialists | journal articles, personal communication with the trialists, unpublished aggregate data | journal articles, personal communication with the trialists | journal articles, personal communication with the trialists, anonymised data extract provided by trialists | journal articles, personal communication with the trialists, trial protocol, original data | journal articles, personal communication with the trialists, trial protocol, original data | |
| Randomization process | 1.1 allocation sequence random? | unclear | Yes | Yes | Yes | Yes | Yes | Yes | Yes | Yes | |
|  | 1.2 concealed allocation sequence until participants were recruited and assigned to interventions? | unclear | unclear | Yes | Yes | Yes | Yes | Yes | Yes | Yes | |
|  | 1.3 baseline imbalances that suggest a problem with the randomization process? | Yes: strong group differences in maternal smoking ‎during pregnancy -which is a strong effect modifier – ‎‎(De Jong Study, Groningen). It was not adjusted for ‎because it was not an a-priori risk factor.‎ | No published baseline characteristics at randomisation | No | No | No | No | No | N | No | |
|  | 1. randomisation risk | high | high/ missing info | low | low | low | low | low | low | low | |
| Deviations from intended interventions | 2.1 participants aware of their assigned intervention during the trial? | unclear | No | Unclear, see page 153/54 of: https://doi.org  ‎/10.1093/ajcn/85.1.152‎ | No | No | No | No | No | No | |
|  | 2.2 carers and trial personnel aware of participants' assigned intervention during the trial? | No | No | No | No | No | No | No | No | No | |
|  | 2.3 If YES/PARTLY YES/ NO INFORMATION to 2.1 or 2.2: Were there deviations from the intended intervention beyond what would be expected in usual practice? | - | - | - | - | - | - | - | - | - | |
|  | 2.4 If Y/PY to 2.3: Were these deviations from intended intervention unbalanced between groups and likely to have affected the outcome? | - | - | - | - | - | - | - | - | - | |
|  | 2.5 any participants analysed in a group different from the one to which they were assigned? | No | No | No | No | No | No | No | No | No | |
|  | 2.6 If YES/PARTLY YES/ NO INFORMATION to 2.5: Was there potential for a substantial impact (on the estimated effect of intervention) of analysing participants in the wrong group? | - | - | - | - | - | - | - |  |  | |
|  | 2. deviation from intervention risk | Unclear/ missing info | low | Unclear/ missing info | low | low | low | low | low | low | |
| Missing outcome data | 3.1 outcome data available for all, or nearly all, participants randomized? *number followed up over number randomised irrespective of whether it was only planned to follow up participants who completed the intervention or took part in a previous follow up because any limitation to the original sample size can introduce bias. | 72/91 (79.1%) | unclear | 184/309 (59.5%) | 52/79 (65.8%) | 214 out of 314 (68.2%) | 42/92 (45.7%) | 30/80 (37.5%) | 17/ 196 (8.7%) | 107/ 128 (45%) | |
|  | 3.2 If NO/PARTLY NO/ NO INFORMATION to 3.1: Are the proportions of missing outcome data and reasons for missing outcome data similar across intervention groups? | unclear | unclear | Yes | Yes | Selective attrition higher proportion (26% vs 18%) lost to follow up in LCPUFA group vs control | unclear | Yes | Yes | No: higher maternal education in LCPUFA group compared to control group | |
|  | 3.3 If NO/PARTLY NO/ NO INFORMATION to 3.1: Is there evidence that results were robust to the presence of missing outcome data? | unclear | unclear | Yes: MI and CC give the same results | unclear | Authors discuss this might lead to underestimation of negative effect. | unclear | Yes | No | Adjustment for imbalances changed (published) results. Adjusted results were used in this systematic review. | |
|  | 3. missing outcome data risk | high | high/ missing info | high | high | high | high | high | high | high | |
| Measurement of the outcome | 4.1 outcome assessors aware of the intervention received by study participants? | unclear | No | No | No | No | unclear | No | No | No | |
|  | 4.2 If YES/PARTLY YES/ NO INFORMATION to 4.1: Was the assessment of the outcome likely to be influenced by knowledge of intervention received? | unclear | - | - | - | - | unclear | - | - | - | |
|  | 4. outcome measurement risk | Unclear/ missing info | low | low | low | low | Unclear/ missing info | low | low | low | |
| Selection of the reported result | 5.1 reported outcome data likely to have been selected from multiple outcome measurements within the outcome domain? | No | Yes: multiple scales of receptive vocabulary (without mentioning which was the intended primary outcome) | No | No | No: outcome is based on (unpublished) data provided by the trialists | Yes: raw and standardised scores at two different ages and different scales without info on what the intended primary outcome was | No: outcome is based on (unpublished) data provided by the trialists | No: outcome is based on (unpublished) data provided by the trialists | No: outcome is based on (unpublished) data provided by the trialists | |
|  | 5.2 reported outcome data likely to have been selected from multiple analyses of the data? | No | unclear | No | No | No | Yes: combined and separate intervention levels unclear which one was the intended primary outcome | No | No | No | |
|  | 5. selection bias risk | low | high | low | low | low | high | low | low | low | |
| Abbreviations: SCT Scotland ENG England WPPSI WASI PPVT | | | | | | | | | | |  |

**S3 Table. GRADE Summary of Findings**

| LCPUFA supplemented baby milk formula compared to unsupplemented baby milk formula for long term cognitive ability | | | | |
| --- | --- | --- | --- | --- |
| Patient or population: term and preterm infants - long term cognitive ability  Setting: hospital and community  Intervention: LCPUFA supplemented infant formula  Comparison: Infant formula without LCPUFA supplementation | | | | |
| Outcomes | № of participants (studies) Follow-up | Certainty of the evidence (GRADE) | Anticipated absolute effects | |
|  |  |  | Risk with unsupplemented infant formula | Risk difference (fixed effects) with LCPUFA supplemented infant formula |
| Full scale IQ in children born at term (WPPSI-R) assessed with: Wechsler Preschool and Primary Scales of Intelligence-R follow up: range 4 years to 4.5 years | 395 (4 RCTs) | ⨁⨁◯◯ LOW ^a^ | The mean full scale IQ in children born at term ranged from 89.9 to 109.7 IQ points | MD 2.50 IQ points lower (5.26 lower to 0.27 higher) |
| Full scale IQ in children born preterm (WASI IQ )  assessed with: Wechsler Adult Scale of Intelligence  follow up: range 10 years to 16 years | 124 (2 RCTs) | ⨁⨁◯◯ LOW ^a^ | The mean full scale IQ in children born preterm ranged from 93.9 to 103.2 IQ points | MD 1.88 IQ points lower (6.04 lower to 2.29 higher) |
| Overall Cognitive ability in term-born children  assessed with: latest available cognitive outcome follow up: range 3.3 years to 16 years | 579 (6 RCTs) | ⨁⨁◯◯ LOW ^a^ | - | SMD 0.10 SD lower (0.32 lower to 0.12 higher) |
| *The risk in the intervention group (and its 95% confidence interval) is based on the assumed risk in the comparison group and the relative effect of the intervention (and its 95% CI).  CI: Confidence interval; MD: Mean difference | | | | |
| GRADE Working Group grades of evidence High certainty: We are very confident that the true effect lies close to that of the estimate of the effect Moderate certainty: We are moderately confident in the effect estimate: The true effect is likely to be close to the estimate of the effect, but there is a possibility that it is substantially different Low certainty: Our confidence in the effect estimate is limited: The true effect may be substantially different from the estimate of the effect Very low certainty: We have very little confidence in the effect estimate: The true effect is likely to be substantially different from the estimate of effect | | | | |

#### Explanations

a. Downgraded 2 levels Reasons: high rate of attrition, high statistical heterogeneity, indication of publication bias

**S4 Table. Medline PubMed Search Strategy**

| ((("fatty acids, omega-3"[MeSH Terms] OR ("fatty"[TIAB] AND "acids"[TIAB] AND "omega-3"[TIAB]) OR "omega-3 fatty acids"[TIAB] OR ("n3"[TIAB] AND "fatty"[TIAB] AND "acid"[TIAB]) OR "n3 fatty acid"[TIAB]) OR (n6[TIAB] AND ("fatty acids"[MeSH Terms] OR ("fatty"[TIAB] AND "acids"[TIAB]) OR "fatty acids"[TIAB] OR ("fatty"[TIAB] AND "acid"[TIAB]) OR "fatty acid"[TIAB])) OR ("fatty acids, unsaturated"[MeSH Terms] OR ("fatty"[TIAB] AND "acids"[TIAB] AND "unsaturated"[TIAB]) OR "unsaturated fatty acids"[TIAB] OR ("polyunsaturated"[TIAB] AND "fatty"[TIAB] AND "acid"[TIAB]) OR "polyunsaturated fatty acid"[TIAB]) OR ("fish oils"[MeSH Terms] OR ("fish"[TIAB] AND "oils"[TIAB]) OR "fish oils"[TIAB]) OR ("docosahexaenoic acids"[MeSH Terms] OR ("docosahexaenoic"[TIAB] AND "acids"[TIAB]) OR "docosahexaenoic acids"[TIAB] OR "DHA"[TIAB]) OR ("arachidonic acid"[MeSH Terms] OR ("arachidonic"[TIAB] AND "acid"[TIAB]) OR "arachidonic acid"[TIAB]) OR (lcpufa[TIAB] OR pufa[TIAB] OR "borage oil"[TIAB] OR "fish oil"[TIAB] OR "evening primrose"[TIAB])) AND ("Infant Formula"[Mesh] OR "Infant Food"[Mesh] OR "Infant Nutritional Physiological Phenomena"[Mesh] OR "follow*on*formula"[TIAB] OR "baby food"[TIAB] OR ("bottle feeding"[MeSH Terms] OR ("bottle"[TIAB] AND "feeding"[TIAB]) OR "bottle feeding"[TIAB]))) AND (("bsid"[TIAB] OR "bayley scale"[TIAB] OR "bayley scales"[TIAB] OR "bayley"[TIAB]) OR ("wechsler scales"[mh] OR ("wechsler"[TIAB] AND "scales"[TIAB]) OR "wechsler scale"[TIAB] OR "wechsler scales"[TIAB]) OR ("brunet-lezine"[TIAB] OR ("brunet"[TIAB] AND "lezine"[TIAB])) OR "fagan test"[TIAB] OR ("stanford binet"[TIAB] OR "stanford-binet"[TIAB]) OR "peabody picture vocabulary"[TIAB] OR ("developmental quotient"[TIAB] OR "development quotient"[TIAB]) OR (("cognitive"[TIAB] AND ("aptitude"[mh] OR ("aptitude"[MeSH Terms] OR "aptitude"[TIAB]) OR "ability"[TIAB] OR "performance"[TIAB])) OR "aptitude tests"[mesh]) OR ("intelligence"[TIAB] OR "intelligence"[mh] OR "intellect"[TIAB]) OR ("cognition"[mh] OR "cognition"[TIAB] OR "cognitive function"[TIAB] OR ("cognitive"[TIAB] AND "function"[TIAB])) OR ("child development"[mh] OR "neurodevelopment"[TIAB]) OR ("education"[Subheading] OR "education"[TIAB] OR "educational status"[MeSH Terms] OR ("educational"[TIAB] AND "status"[TIAB]) OR "educational status"[TIAB] OR "education"[TIAB] OR "education"[MeSH Terms]) OR ((educational[TIAB] AND "school"[TIAB] OR academic[TIAB]) AND (attainment[TIAB] OR ("achievement"[MeSH Terms] OR "achievement"[TIAB]) OR performance[TIAB]))) AND (randomized controlled trial[pt] OR controlled clinical trial[pt] OR randomized[tiab] OR placebo[tiab] OR "clinical trials as topic"[MeSH Terms:noexp] OR randomly[tiab] OR trial[ti] NOT ("animals"[MeSH Terms] NOT "humans"[MeSH Terms])) Filters: Humans; Child: birth-18 years |
| --- |

**S5 Table. Ovid Embase Search Strategy**

| # | Searches | Results |
| --- | --- | --- |
| 1 | exp omega 3 fatty acid/ or ("fatty" and "acids" and "omega-3").ab,ti. or ("n3" and "fatty" and "acid").ab,ti. | 34660 |
| 2 | exp unsaturated fatty acid/ or ("fatty" and "acids" and "unsaturated").ab,ti. | 146217 |
| 3 | exp polyunsaturated fatty acid/ or "polyunsaturated fatty acid".ab,ti. | 24545 |
| 4 | exp fish oil/ or ((fish or borage or evening primrose) and oil).ab,ti. | 22658 |
| 5 | exp docosahexaenoic acid/ or ("docosahexaenoic" and "acid").ab,ti. or dha.ab,ti. | 29150 |
| 6 | exp arachidonic acid/ or ("arachidonic" and "acid").ab,ti. | 53627 |
| 7 | exp long chain fatty acid/ or (lcpufa or pufa).ab,ti. | 325102 |
| 8 | 1 or 2 or 3 or 4 or 5 or 6 or 7 | 387715 |
| 9 | exp artificial milk/ | 13328 |
| 10 | exp infant nutrition/ | 82701 |
| 11 | exp bottle feeding/ or ("bottle" and "feeding").ab,ti. | 5184 |
| 12 | 9 or 10 or 11 | 83384 |
| 13 | exp Wechsler intelligence scale/ or (Wechsler and scale).ab,ti. | 13291 |
| 14 | exp Bayley Scales of Infant Development/ or (bsid or bayley scale*).ab,ti. | 3706 |
| 15 | exp peabody picture vocabulary test/ or (peabody and picture and vocabulary).ab,ti. | 664 |
| 16 | exp Stanford-Binet Intelligence Scale/ or ("stanford binet" or "stanford-binet").ab,ti. | 607 |
| 17 | (("brunet" and "lezine") or (fagan and test)).ab,ti. | 222 |
| 18 | exp mental development/ or exp psychomotor development/ or developmental quotient.ab,ti. | 35342 |
| 19 | exp aptitude/ or (cognitive and (performance or ability or function)).ab,ti. | 190752 |
| 20 | exp intelligence/ or intellect.ab,ti. or exp cognition/ or neurodevelopment.ab,ti. | 2108363 |
| 21 | exp educational status/ or exp academic achievement/ or exp education/ or ((education* or school or academic) and (attainment or achievement or performance or status)).ab,ti. | 1455309 |
| 22 | 13 or 14 or 15 or 16 or 17 or 18 or 19 or 20 or 21 | 3282367 |
| 23 | crossover-procedure/ or double-blind procedure/ or randomized controlled trial/ or single-blind procedure/ or (random* or factorial* or crossover* or cross over* or placebo* or (doubl* adj blind*) or (singl* adj blind*) or assign* or allocat* or volunteer*).tw. | 2204572 |
| 24 | limit 23 to human | 1728320 |
| 25 | 23 and 24 | 1728320 |
| 26 | 8 and 12 and 22 and 25 | 148 |

* Results last updated in October 2019.

**S6 Table. Contacts with Trialists**

| Study | Queries | Contacts | Items remaining unresolved |
| --- | --- | --- | --- |
| Term Kansas, Oregon, Portland (1992-93) | Time of recruitment, was analysis intention to treat or per protocol; What was the reasoning for not adjusting for imbalances in maternal smoking during pregnancy? Aware of any other RCTs? | Bridget Barrett-Reis, Abbott (emails to: 09. Nov 2018, 22. Nov 2018 \| replies: 23. Nov 2018)  Geraldine Baggs, Abbott (replies: 27. Dec 2018) Nancy Auestad (emails to: 09. Nov 2018\| replies: 09. Nov 2018) | none |
| Term Europe 6 centre trial (1992-93) | Was this a 6 centre trial with a single randomisation protocol?; Number of children originally randomised, identity of remaining two centres and investigators, baseline characteristics of children at randomisation vs follow-up; Follow-up numbers are not consistent across publications, year the study was conducted; Aware of any other RCTs? | Tayside Ethics (emails to: 06. Feb 2018 \| replies: 06. Feb 2018 )  Peter Willats (emails to: 08. Feb 2018, 12. Feb 2018, 26. Feb 2018, 28. Sep 2018 \| replies: 12. Feb 2018)  Stewart Forsyth (emails to: 01. Feb 2018 \| replies: 02. Feb 2018 ) | Was this a 6 centre trial with a single randomisation protocol?, Number of children originally randomised, identity of remaining two centres and investigators, baseline characteristics of children at randomisation vs follow-up, Follow-up numbers are not consistent across publications, |
| Term NottinghamLeicester  (1993-95) | n/a | Individual level data available | n/a |
| Term Dallas, TX (1993-95) | Was the allocation sequence ‎concealed until the participants were assigned to the ‎interventions?‎ Were the analyses based on intention-to-treat or per-protocol? p.280 of the 2007 pub by birch et al and table 1 seem to suggest per-protocol (i.e. analyses based on adherence rather than assignment to intervention). | Eileen Birch (emails to: 25. Jan 2019, \| replies: 25. Jan 2019) | none |
| Term Groningen (1997-99) | Request to send analysis results without interaction term (smoking), Aware of any other RCTs? Was the allocation sequence concealed until participants were recruited and assigned to interventions? A higher proportion (26% vs 18%) was lost ‎to follow up in LCPUFA group vs ‎control group when the WASI was measured. Is there evidence that results were robust to the presence of missing outcome data? When the WASI was measured -were the psychologists blind to the type of formula feeding the infant had received? (The 2012 paper only mentions that examiners were blind at the 18 months measurement) | Mijna Hadders-Algra (emails to: 19. Jan 2018, 21 Jan 2019 \| replies: 23. Jan 2018, 29.01.2019) | none |
| Term Dallas/ Kansas DIAMOND Study (2002-04) | Request to send analysis results as numbers (publication only contained figure), The funding number does not match up with the research protocol, Aware of any other RCTs?  How many children were initially enrolled in your study by group and study site? The follow-up publications only mention the number who completed the intervention and do not add up to N=343 which is the number in the initial publication but I am unable to find it broken down by centre and group and therefore cannot calculate the follow-up rate for the different outcomes based on enrolment. I do understand that three participants were enrolled who did not meet the protocol inclusion criteria and were included in the statistical analyses – which centre did they belong to?  In Kansas: were the proportions of missing outcome data and reasons for missing outcome data similar across intervention groups in the cognitive follow-up study (PPVT and BBCS-R, Drover 2012) In Kansas: were the outcome assessors for the PPVT and BBCS-R aware of the intervention received by the study participants? In Dallas: were the proportions of missing outcome data and reasons for missing outcome data similar across intervention groups in the cognitive follow-up study (WPPSI, Colombo 2017) In Dallas: were the outcome assessors for the WPPSI aware of the intervention received by the study participants? | Susan Carlson (emails to: 23. Aug 2018, 25. Jan 2019 \| replies: 23. Aug 2018, 27. Jan 2019)   John Colombo (emails to: 17. Jan 2018 \| replies: 17. Jan 2018) | none |
| Term NottinghamLeicester (1993-96) | n/a | Individual level data available | n/a |
| Term Glasgow (1995-97) | n/a | Individual level data available | n/a |
| Other | Aware of any (potentially unpublished) cognitive follow-ups from LCPUFA RCTs? | Makrides M. (emails to: 29. Jan 2018 \| replies: 30. Jan 2018)  Lauritzen L. (emails to: 29. Jan 2018, 02. Feb 2018 \| replies: 31. Jan 2018, 03. Feb 2018)  Jelinek J. (emails to: 08. Aug 2018, 28. Sep 2018, 02. Oct 2018, 03. Oct 2018 \| replies: 02. Oct 2018, 03. Oct 2018)  Fewtrell M. (emails to: 28. Jan 2018 \| replies: 28. Jan 2018)  AOCS Conference admin (emails to: 03. Oct 2018 \| replies: none)  Virgilio P. Carnielli (emails to: 15. Nov 2019)  Clandinin T. (emails to: 15. Nov 2019 \| replies: 17.Nov 2019)  Fang P. (emails to: 15. Nov 2019)  Lapillonne A. (emails to: 15. Nov 2019 \| replies: 15. Nov 2019) | n/a |

**S7 Table. Potential Conflicts of Interest**

| Study | All co-authors on publications of cognitive outcomes | Nature of potential COI | Patents for use of DHA in infant formula | Years cognitive outcomes* were published | Funder of original trial / provider of study formula | Funder(s) for cognitive follow-up studies reported in this manuscript |
| --- | --- | --- | --- | --- | --- | --- |
| Term US: 3 centres‎ ‎(1992-93)‎ | Auestad Nancy, Scott David T, Janowsky Jeri, Jacobsen Cynthia, Carroll Robin E, Montalto Michael B, Halter Robin, Qiu Wenzi, Jacobs Joan R, Connor William E, Connor Sonja L, Taylor J, Neuringer Martha, Fitzgerald-Gustafson KM, Hall Robert T | Several authors (Auestad N, Janowsky J, Halter R, Fitzgerald-Gustafson KM, Neuringer M, Montalto MB) held patents for DHA infant formulas at the time of publication, several authors were employed by Ross at the time of publication (Auestad N, Monalto MB, Halter RMA, Qiu W, Jacobs JR) | US20020045660-A1 (Infant formulas containing long-chain polyunsaturated fatty acids and uses thereof, 2001) US20030190363-A1 (Infant formulas containing long-chain polyunsaturated fatty acids and uses thereof, 2001) | 1997, 1998, 2003 | Ross (Abbott Laboratories) | Ross (Abbott Laboratories) |
| Term Europe: 6 centres‎ (1992-93) | Willats Peter, Forsyth J Steward, DiModugno MK, Varma S, Colvin M, Casaer Paul, Agostoni Carlo, Bruzzese Maria Grazia, Trojan Sabina, Bellu Roberto, Riva Enrica, Bissenden J , Smith M, Elliot A, Eggermont Ephrem, McNaughton A, Boehm Günther | Boehm G (senior author) was employed by Danone (NUMICO) at the time the trial was conducted and held patents for DHA infant formulas at the time of publicaton, the trial was also designed by industry | WO2010110658-A1 + WO2010110649-A1 (The present invention concerns a kit of parts of infant milk formula comprising different amount of DHA for stimulating the development of brains, 2009) EP1656839-A1 (Nutrition containing lipid blend, 2004) | 1995, 1997, 1998, 2003, 2013 | Danone  (Numico) | Danone  (Numico) |
| Term ENG: 2 centres (1993-95) | Lucas Alan, Stafford Mai, Abbott Rebecca, Stephenson Terence, MacFayden Una, Elias-Jones Alun, Clements Helena |  |  | 1999 | Nestlé (Nestec Ltd.) | Medical Research Council (MRC) and EU framework 6 grant (EARNEST) |
| Term US: Dallas ‎(1993-95) | Birch Eileen, Garfield Sharon, Castaneda Y, Hughbanks-Wheaton D, Hoffman Dennis R, Uauy Ricardo, Birch David G | Several authors (Birch E, Hoffman DR) held patents for DHA infant formulas at the time of publication | US20020045660-A1 (Infant formulas containing long-chain polyunsaturated fatty acids and uses thereof. Methods for providing nutrition and for enhancing neurological development of preterm infants are disclosed, 2001) US7413759-B2 (Method of enhancing cognitive ability in infant fed DHA containing baby-food compositions, 1998) | 1998, 2000, 2007 | Mead  Johnson (Enfamil) | National Institutes of Health (NIH) |
| Term NL: Groningen ‎(1997-99) | De Jong Corina, Kikkert Hedwig, Fidler Vaclav, Hadders-Algra Mijna, Bouwstra H, Dijck-Brouwer D, Wildeman JA, Tjoonk HM, van der Heide JC, Boersma ER, Muskiet FA, Boehm Günther | Boehm G was employed by Danone (NUMICO) at the time the trial was conducted and held patents for DHA infant formulas, Boehm G also reviewed drafts of follow-up publications | WO2010110649-A1 + WO2010027258-A1 (The present invention concerns a kit of parts of infant milk formula comprising different amount of DHA for stimulating the development of brains, 2009) EP1656839-A1 (Nutrition containing lipid blend, 2004) | 2003, 2005, 2010, 2012 | Danone  (Numico) | EU framework 6 grant (EARNEST) |
| Term Dallas/ Kansas DIAMOND Study (2002-04) | Birch Eileen, Colombo John, Carlson, Susan E, Cheatham CL, Castaneda YS, Doty T, Diersen-Schade Deborah A, Drover James R, Fu VL, Fitzgerald-Gustafson KM, Hoffman Dennis R, Kepler A, Kerling EH, Liao K, Lepping RJ, Minns L, Mundy D, Marunycz, McCandliss BD, Sittiprapaporn W, Shaddy DJ, Wheaton DK | Several authors (Birch E, Carlson SE, Fitzgerald-Gustafson KM, Hoffman DR, Diersen-Schade D) hold patents for DHA infant formulas, additionally Drs. Hoffman and Birch are employed by the Retina Foundation which is funded by Mead Johnson | US-9375028-B2 (Compositions and methods for nutrient delivery, 2010) US20020045660-A1 (Infant formulas containing long-chain polyunsaturated fatty acids and uses thereof - Methods for providing nutrition and for enhancing neurological development of preterm infants are disclosed, 2000) US7413759-B2 (Method of enhancing cognitive ability in infant fed DHA containing baby-food compositions, 2004) | 2010, 2011, 2017 | Mead  Johnson (Enfamil) | Mead Johnson Nutrition,  NIH, Kansas Intellectual and Developmental Disabilities Research Center |
| Preterm ENG: ‎2 centres (1993-96) | Lucas Alan, Fewtrell Mary, Morley Ruth, Abbott R, Singhal A, Isaacs EB, Stephenson T, MacFayden U |  |  | 2002 | Danone  (Numico) | EU framework 6 grant (EARNEST) |
| Preterm SCT: Glasgow ‎(1995-97) | Fewtrell M, Abbott R, Kennedy K, Singhal A, Morley R, Caine Eleanor, Jamieson EC, Cockburn F, Lucas A, Weaver L, Ross S, Isaacs EB | Weaver is a member of the Infant and Toddler Forum, an educational charity funded by Danone |  | 2004, 2011 | Heinz | EU framework 6 grant (EARNEST) |

* This concerns cognitive outcomes < age 2.5 years as well as ≥ age 2.5 years

S1 Fig. SMDs from all included cognitive outcomes in previously published (hollow) and unpublished (coloured) trials, ‎comparing LCPUFA-‎supplemented vs unsupplemented infant formula in term (circles) and preterm (crosses) populations, against their ‎standard error.‎


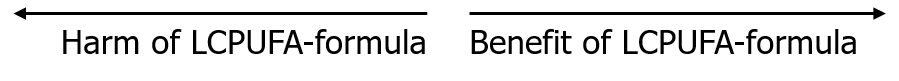

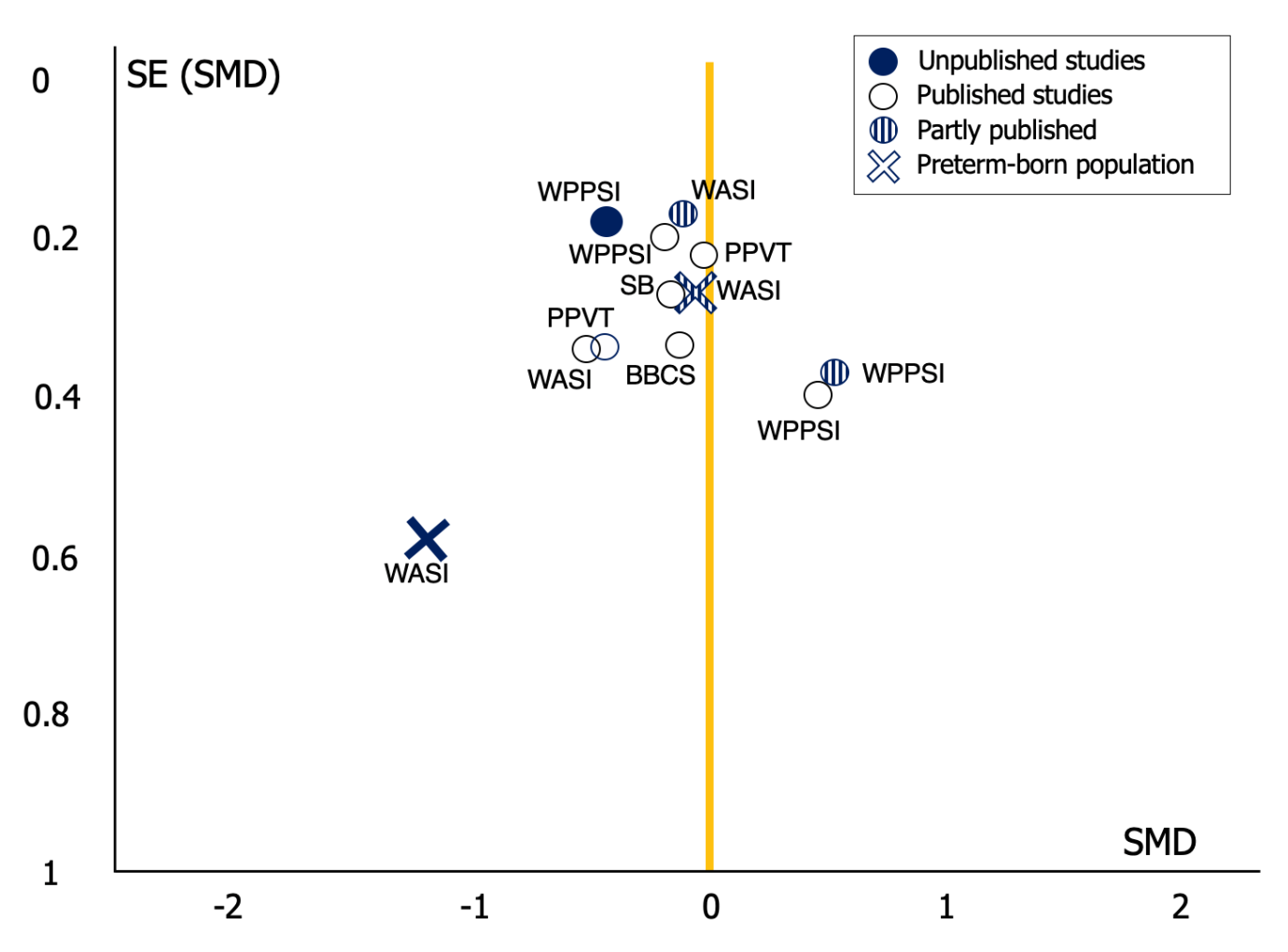


S2 Fig. WPPSI-R IQ in terms age 4-6 years


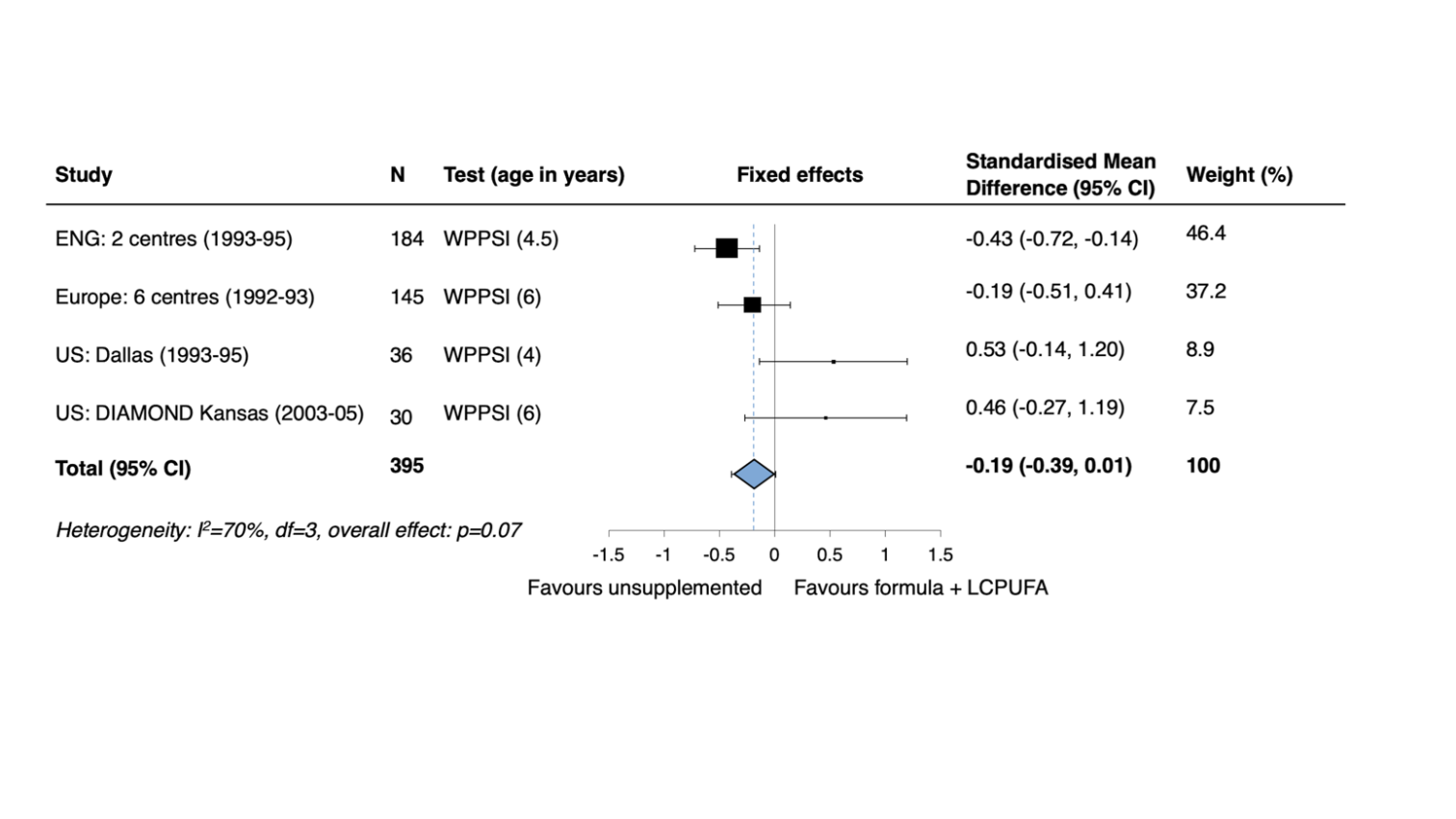


S3 Fig. WASI IQ in preterms age 10-16 years

**
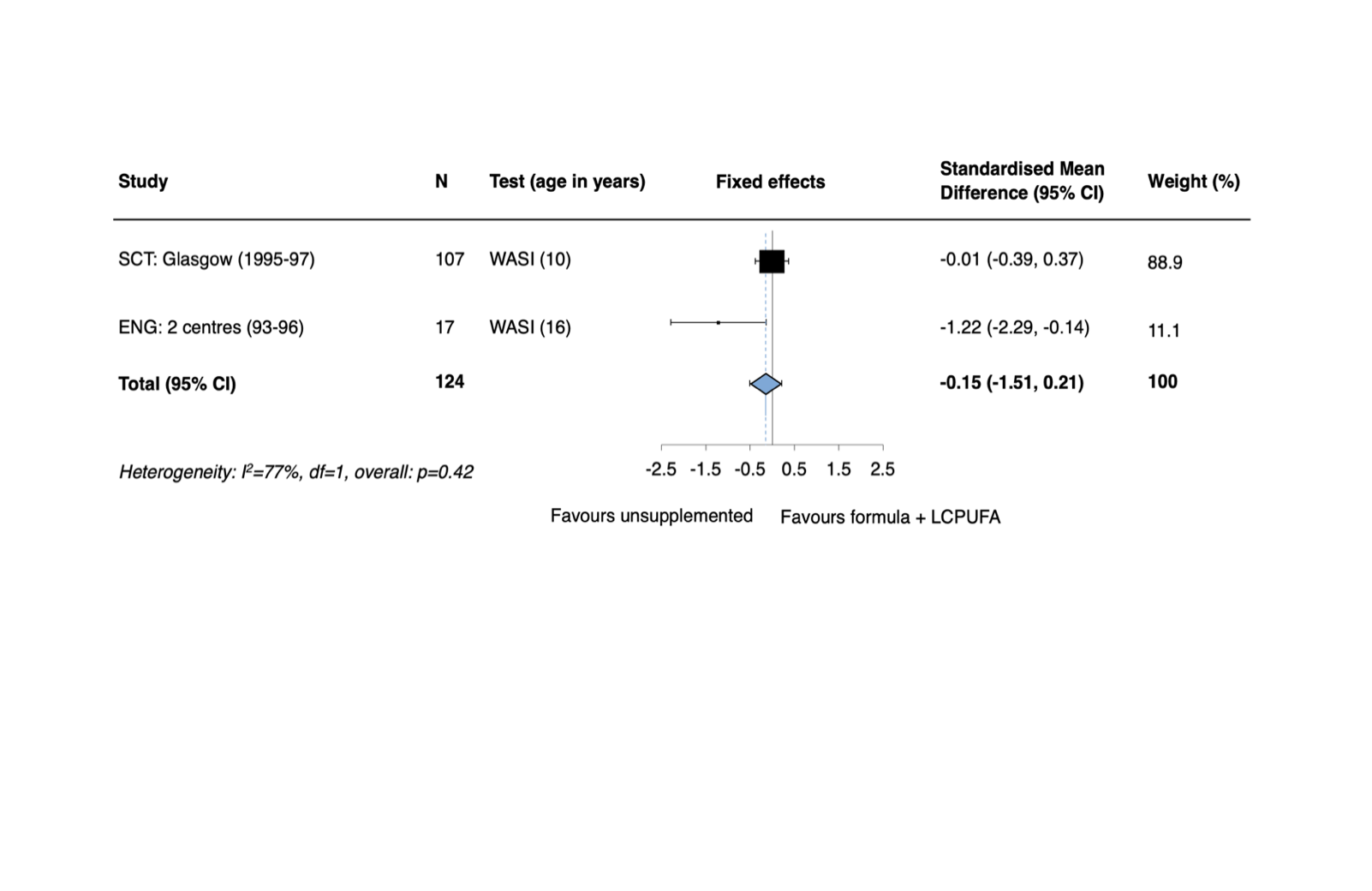
**

**S4 Fig. WASI IQ in terms age 9-16 years**


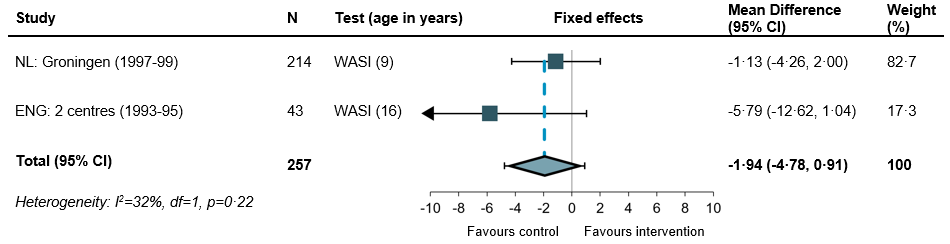


**S5 Fig. Peabody Picture Vocabulary Test in terms age 3-4 years**


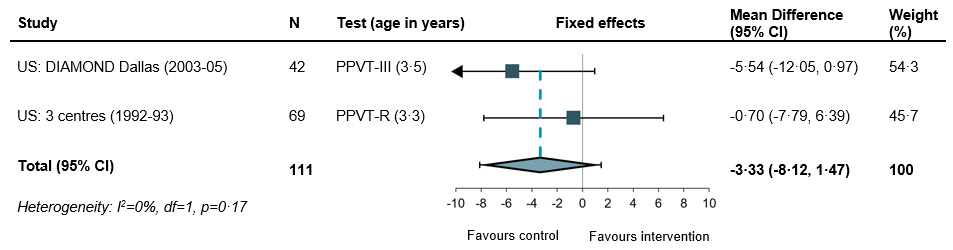


**S6 Fig. Stanford-Binet IQ in terms age 9 years**


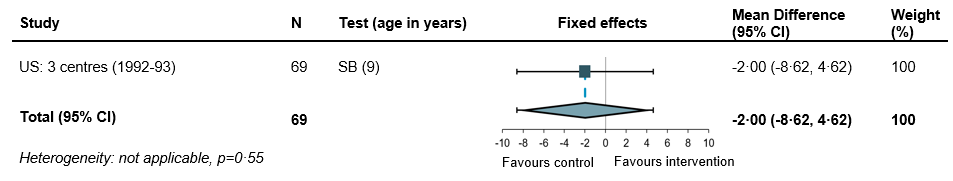


**S7 Fig. Bracken Basic Concept Scale School Readiness in terms age 2‎.5 years**


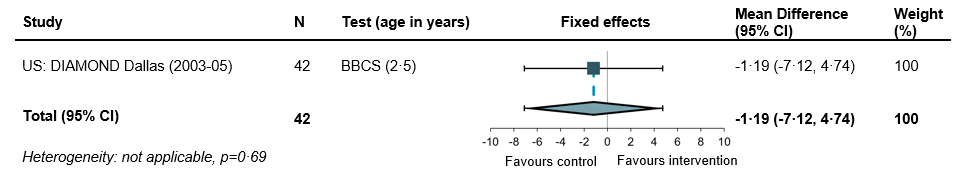


**References**

AUESTAD, N., SCOTT, D. T., JANOWSKY, J. S., JACOBSEN, C., CARROLL, R. E., MONTALTO, M. B., HALTER, R., QIU, W., JACOBS, J. R., CONNOR, W. E., CONNOR, S. L., TAYLOR, J. A., NEURINGER, M., FITZGERALD, K. M. & HALL, R. T. 2003. Visual, cognitive, and language assessments at 39 months: a follow-up study of children fed formulas containing long-chain polyunsaturated fatty acids to 1 year of age. Pediatrics, 112, e177-83.

BIRCH, E. E., GARFIELD, S., CASTANEDA, Y., HUGHBANKS-WHEATON, D., UAUY, R. & HOFFMAN, D. 2007. Visual acuity and cognitive outcomes at 4 years of age in a double-blind, randomized trial of long-chain polyunsaturated fatty acid-supplemented infant formula. Early Hum Dev, 83, 279-84.

DROVER, J. R., FELIUS, J., HOFFMAN, D. R., CASTANEDA, Y. S., GARFIELD, S., WHEATON, D. H. & BIRCH, E. E. 2012. A randomized trial of DHA intake during infancy: school readiness and receptive vocabulary at 2-3.5 years of age. Early Hum Dev, 88, 885-91.

WILLATTS, P., FORSYTH, S., AGOSTONI, C., CASAER, P., RIVA, E. & BOEHM, G. 2013. Effects of long-chain PUFA supplementation in infant formula on cognitive function in later childhood. Am J Clin Nutr, 98, 536S-42S.
