# Supplementary material for: Effect of long-chain polyunsaturated fatty acids in infant formula on long-term cognitive function in childhood: A systematic review and meta-analysis of randomised controlled trials
Source: PLoS One. 2020 Nov 5;15(11):e0241800. doi: 10.1371/journal.pone.0241800 (PMC7644261; doi:10.1371/journal.pone.0241800)
Supplement: Attachment — Submitted filename: 2020-09-07 Response to reviewers.docx [file pone.0241800.s003.docx]

Response to reviewers

Manuscript ID: PONE-D-20-14949

*7 Sept 2020*

**Response to reviewer 1:**

1. Is the manuscript technically sound, and do the data support the conclusions? ***Yes***

2. Has the statistical analysis been performed appropriately and rigorously? ***Yes***

3. Have the authors made all data underlying the findings in their manuscript fully available? ***Yes***

4. Is the manuscript presented in an intelligible fashion and written in standard English? ***Yes***

5. Comments to the Author:

*The authors performed a systematic review and meta-analysis of RCTs to assess the effect of LCUFA on long-term cognitive function in childhood. The authors concluded that “there was no evidence of benefit of LCPUFA supplementation in term or preterm-born infants and weak evidence that LCPUFA reduced IQ score in term-born children.” Although the overall approach to the review is proper, I have few comments:*

| Nr | Comment | Response | Location of change |
| --- | --- | --- | --- |
| **1.1** | *I would recommend adding the software used for analysis.* | We added the software used for the analysis to the abstract | **Abstract** |
| **1.2** | *Please place the keywords after the abstract.* | Done | **Abstract** |
| **1.3** | *The research question was clearly outlined, and the research question justified given what is already known about the topic.* | Thank you, we appreciate the positive feedback. | **No change** |
| **1.4** | *The text starts with references [3-5]. References should be numbered in consecutive order in the main text starting from “Introduction.”* | References 1-2 were placed behind the abstract to refer to the study protocols. We now removed these references so that the introduction starts with reference 1. | **References p.5** |
| **1.5** | *The methodology described in the study seems to have followed current standardized procedures and guidelines for systematic review and meta-analysis. The methods are detailed enough to allow replication of the analysis. Outcomes are well-defined; the same is true for quality assessment and data analysis.* | Thank you, we appreciate the positive feedback. | **No change** |
| **1.6** | *Page 5: please define the PRISMA guidelines “Preferred Reporting Items for Systematic Reviews and Meta-Analyses.”* | Done | **Search strategy p.6** |
| **1.7** | *Page 6: The paragraph starting with “The secondary outcomes…”, delete “standardised mean difference” because it is previously defined.* | Done | **Methods p.7** |
| **1.8** | *“We included eight unique trial cohorts,” please add references.* | Done | **Results p.7** |
| **1.9** | *a comma should be placed between the effect size and the 95% Cl.* | Done | **Primary outcome p.10** |
| **1.10** | *The results are discussed from multiple angles and placed into context without being overinterpreted, and conclusions answer the aims of the study.* | Thank you for the positive feedback | **No change** |
| **1.11** | *The “Cochrane Central Register” search results are missing.* | Thank you for pointing out this oversight, we have updated the figure | **Figure 1** |
| **1.12** | *49 full-text articles assessed, the authors excluded 40 articles (27+13); thus, 9 articles should be retrieved, not 8. Please recheck!!* | 8 is correct, it refers to the trial cohorts rather than the trial publications. | **No change** |

**Response to reviewer 2:**

1. Is the manuscript technically sound, and do the data support the conclusions? ***Yes***

2. Has the statistical analysis been performed appropriately and rigorously? ***No***

3. Have the authors made all data underlying the findings in their manuscript fully available? ***Yes***

4. Is the manuscript presented in an intelligible fashion and written in standard English? ***No***

5. Comments to the Author:

| Nr | Comment | Response | Location of change |
| --- | --- | --- | --- |
| **2.1** | *Please follow the guidelines of the journal (https://journals.plos.org/plosone/s/submission-guidelines)* |  | **Abstract** |
| **2.2** | *References 1 and 2 are missing.* | References 1-2 were placed behind the abstract to refer to the study protocols. We now removed these references so that the introduction starts with reference 1. | **Introduction p.5** |
| **2.3** | *Please add reference for this information "Human breast milk contains DHA, AA, and their fatty acid precursors"* | Done | **Introduction p.5** |
| **2.4** | *Define this abbreviation "IQ" at the first mention.* | Done | **Introduction p.5** |
| **2.5** | *Section of Methods need to be rearranged; you can develop a new sub-section for inclusion and exclusion criteria.* | We included a sub-heading for inclusion and exclusion criteria and have re-arranged the method sections to be more clear. | **Methods p.6** |
| **2.6** | *"Patient involvement" No need for this section or you can move it before the references.* | We removed this section | **Methods** |
| **2.7** | *Please move this section to be before the references "Role of the funding source"* | Done | **Role of the funding source** |
| **2.8** | *"We obtained previously unpublished outcome data for two RCTs: Firstly, a two-centre trial of term babies in England18 19 provided unpublished follow-up data on IQ assessments using the Wechsler Preschool and Primary Scales of Intelligence-Revised (WPPSI-R) at age 4.5 years and the Wechsler Abbreviated Scales of Intelligence (WASI) at age 16 years. Secondly, a two-centre trial in England of babies born preterm provided unpublished data from their IQ assessments using the WASI at age 16 years" Is their any duplication here? Please rephrase to avoid any confusion.* | It is not clear to us what the reviewer means by “*Is their any duplication here? Please rephrase to avoid any confusion.”*  We believe this is an important detail and critical new data that this study seeks to publish. | **No change** |
| **2.9** | *Please mention the Study ID (last name and year) in the table 1.* | A study ID based on last name and year is not applicable for this review because we base our analyses on trial cohorts rather than publications - mainly because not every outcome was published. | **No change** |
| **2.10** | *Remove any question mark in the table 1 and replace it with NA or NR.* | Done | **Table 1** |
| **2.11** | *In Figure 1: Please add additional arms for cochrane library and unpublished data* | Thank you for noticing the oversight regarding the Cochrane library – an arm for unpublished data was already included | **Figure 1** |
| **2.12** | *Did you used R program or Review Manager as these figures not belong to Review Manager?* | We used review manager for the analysis and Excel for creating the figures | **Figure 1** |
| **2.13** | *Try to perform a sensitivity analysis to overcome this heterogeneity* | Due to the low number of included trials it was not possible to explore reasons for heterogeneity. This is now being discussed more clearly in the discussion section. | **Discussion p.12 and 13** |
| **2.14** | *The discussion is very week and need to be supported with some clinical aspects.* | Reviewer 1 remarked “*The results are discussed from multiple angles and placed into context without being overinterpreted, and conclusions answer the aims of the study.”* and reviewer 4 remarked that the discussion is “*Well-written and comprehensive.”* We would appreciate more specific, actionable suggestions from reviewer 2 to improve the discussion to his/her standard. | **No change** |

**Response to reviewer 3:**

1. Is the manuscript technically sound, and do the data support the conclusions? ***Yes***

2. Has the statistical analysis been performed appropriately and rigorously? ***N/A***

3. Have the authors made all data underlying the findings in their manuscript fully available? ***Yes***

4. Is the manuscript presented in an intelligible fashion and written in standard English? ***Yes***

5. Comments to the Author:

*This is a systematic review and meta-analysis in which the authors investigated the long-term cognitive function effects of long-chain polyunsaturated fatty acids in infant formula in childhood at the age of 2.5 years or older. Although the study is well-written and structured, the overall quality of evidence for the included RCTs is low due to heterogeneity and quality of included studies. Moreover, many studies have investigated that topic extensively with similar results. I do not think that this paper will add more information to the medical literature related to that topic.*

Author response:

We thank the reviewer for their comment but respectfully disagree and argue that our findings remain important, even in the context of previous reviews. There are three unique contributions from our study: 1) The inclusion of 6 unpublished or partly published cognitive outcomes from 5 trial cohorts. This data indicates that trial follow-ups suggesting harmful effects from LCPUFA-supplementation are under-represented in current scientific literature, although we are unable to comment on the reasons for this. 2) To the best of our knowledge this is the first systematic review meta-analysing long-term cognitive outcomes after age 2.5 years. In contrast to early developmental outcomes such as the Bayley Scales of Infant and Toddler Development, outcomes at a later age are likely to be better at differentiating cognitive ability levels and are more predictive of future academic success and employment opportunities. 3) Finally, this review provides important and timely evidence for policy decision. For instance, for the European Food Safety Authority (EFSA) who were not able to consider signals of harm relating to LCPUFA-supplementation from the unpublished literature in their previous scientific opinion to the European Commission. Publication of this manuscript would allow the long overdue unpublished data it contains to augment the scientific literature.

**Response to reviewer 4:**

1. Is the manuscript technically sound, and do the data support the conclusions? ***Partly***

2. Has the statistical analysis been performed appropriately and rigorously? ***No***

3. Have the authors made all data underlying the findings in their manuscript fully available? ***Yes***

4. Is the manuscript presented in an intelligible fashion and written in standard English? ***Yes***

5. Comments to the Author:

*The authors conducted a meta-analysis to evaluate long-term effects of long-chain polyunsaturated fatty acids on IQ beyond 2.5 years of age. The findings are interesting and consistent with the previous literature. Some comments warrant attention:*

| Nr | Comment | Response | Location of change |
| --- | --- | --- | --- |
| **4.1** | *Why fixed effect model despite obvious heterogeneity?* | We thank the reviewer for highlighting our reasoning about choosing the fixed effects model over the random effects model. Based on the protocol we took the a-priori decision to perform a fixed effects meta-analysis. The trials included in each meta-analysis were conceptually homogeneous. There were differences in source and dose but sampling error is a plausible cause of heterogeneity, especially as the two smaller studies that report positive effects have similar LCPUFA doses and sources to the studies showing effects in the opposite direction. Further, given the small number of trials in each meta-analysis, a random effects model is likely to produce unstable results. In addition the *I^2^* can be biased in small meta-analyses [1] and should therefore be interpreted with caution. Our approach reflects the previous Cochrane systematic reviews on LCUFA supplementation of infant formula which used fixed effects models throughout. However, we appreciate the reviewers concern about existing heterogeneity and as this has been mentioned by several reviewers we have now included the random effects meta-analyses in the main text. This doesn’t change the conclusion of the study, namely that there is no evidence of benefit of LCPUFA for cognition. | **Abstract, methods and results** |
| **4.2** | *Add the number of randomized children in the results.* | This is not available since one study did not disclose the number of randomised infants. We now added more information about this in Table 1. | **Table 1** |
| **4.3** | *weak evidence of lower IQ in the supplemented group: But the 95% CI is not consistent with this interpretation. It crosses the null value of zero for a quantitative MD.* | We have now removed this sentence. | **Abstract** |
| **4.4** | *A single meta-analysis was registered on PROPSERO with two numbers!!* | This is incorrect, two meta-analyses were registered: one for terms and one for preterms. Both are combined in this publication. | **No change** |
| **4.5** | *What this study adds/What is already known: These sections are not suitable for PLOS One. It seems the manuscript was submitted using the author instructions of another journal (Similar is the abstract).* | We have now removed this section. | **What this study adds/What is already known** |
| **4.6** | *There is an obvious problem with referencing: Numbers are not organized; many references are old back to 1992.* | We refer to the most recent studies possible. Some of these date back to 1992, but this is unavoidable since the trials in this study were conducted around that time and these are the trials which inform current guidelines. We checked the organisation of references for any inconsistencies and the firs reference in the main text now starts at “1”. | **References throughout manuscript** |
| **4.7** | *Because human breast milk contains LCPUFA, could the authors account for breast-fed children in their analysis?* | It is not quite clear what the reviewer is referring to. This is a meta-analysis of RCTs (no observational studies included) and the comparison is between LCPUFA supplemented vs unsupplemented infant formula. The eligibility criteria for the included trials allowed breast feeding in one trial but as all studies were randomised and mothers were blind to allocation to LCPUFA vs not, the trial design is expected to account for any potential confounding from breast milk consumed feeding by the trial participants. We have now made this more clear in the results. | **Results p.8** |
| **4.8** | *At the start of the third paragraph, the authors should add "To our knowledge, ".* | We added this | **Introduction p.5** |
| **4.9** | *The rationale for the meta-analysis is adequately stated.* | Thank you | **No change** |
| **4.10** | *"proceedings from major scientific meetings of child nutrition" can you specify?* | In particular we contacted organisers of the *AOCS Conference, PUFA in Infant Nutrition: Consensus and Controversies* which generated several relevant abstracts that did not have an associated publication. We specify this in the supplementary material under correspondence with trialists. | **No change, see supplementary material S6 Table: Contacts with Trialists** |
| **4.11** | *I see that the authors sub-grouped according to preterm/term delivery. But are there any data on children with low birth weight?* | The focus of the trials was on term and preterm infants because these might differ in their ability to synthesise LCPUFA de novo. The trials were therefore conducted in groups of term or preterm infants, rather than in groups of infants defined primarily by their birthweight. Within each grouping of term or preterm infants there would be a range of birthweights, although many term studies will have a lower limit and exclude those below 2.5kg for example (see Table 1. Characteristics of included RCT cohorts and associated publications for more details). | **No change** |
| **4.12** | *"using validated measures" which included?* | We have now included examples. | **Methods p.6** |
| **4.13** | *The authors did not provide information on their data extraction process!* | We used the Cochrane risk of bias tool (S2 Table) as data extraction template. We have now clarified this in the text. | **Strength of evidence and risk of bias p.7** |
| **4.14** | *"The primary outcome was the pooled difference, presented as mean difference (MD) and standardized mean difference (SMD), between intervention and control arms, based on the measure of cognitive function reported most frequently among the included studies" this part is not clear!* | Thank you for highlighting the need for revision here, we have now clarified this section in the text. | **Outcomes and data analysis p.7** |
| **4.15** | *So you selected fixed effect model based on previous meta-analyses?* | Please see answer to comment 4.1 | **Abstract, methods and results** |
| **4.16** | *The secondary tests were cognitive test scores?? All IQ scoring tests, because there are various types of cognitive tests?* | Yes, all studies with any cognitive tests were included. The secondary outcomes also include for example the Peabody Picture Vocabulary test which is not a “traditional” IQ test. We now clarified this in the text | **Results p.11 and Figure 4** |
| **4.17** | *The authors used random effects model only for secondary outcome bacause the studies used various tests. But also, the heterogeneity should have been expected for the primary outcome based on other different variables between studies.* | Please see answer to comment 4.1 | **Methods** |
| **4.18** | *Add version and source of RevMan software! These forest plots do not appear to be extracted from RevMan!* | The RevMan version is now specified in the text. The forest plots were generated in Excel version 16 and are based on the RevMan estimates. | **Methods p.7** |
| **4.19** | *Also, specify that ou used the updated Cochrane ROB. II tool.* | We now include the version number of the ROB tool in the text. | **Methods p.7** |
| **4.20** | *The authors evaluated publication bias using funnel plots. They should mention that this method lacks reliability for less than 10 included studies.* | Thank you for the opportunity to discuss how we arrived at the conclusion of potential for selective publication. We did not use the plot as the basis for our reasoning but base our concern primarily on anecdotes from trialists about the (perceived) difficulty of publishing harmful results. We have now moved the plot to the supplementary material and revised our text to state “S1 Figure plots published and unpublished effect estimates against their standard error and indicates that unpublished effect estimates tend to be those that show harm. However, this should be interpreted with caution since visual analysis of potential for publication bias has limited reliability with <10 studies” | **Results p.12** |
| **4.21** | *The authors should comment briefly on ROB assessment results of the included trials.* | Done | **Quality of evidence and risk of bias** |
| **4.22** | *What was the 2.5 year follow-up age cut-off based on?* | The cut-off of ≥2.5 years was based on the age where early development of the prefrontal cortex, the brain region associated with higher cognitive functions is completed [2] We now include this justification in the text. | **Methods p.6** |
| **4.23** | *The authors analyzed based on total IQ scores. But these tests often have sections for different cognitive functions. Could the authors extract any data in this regard?* | This was not part of the review protocol and the small sample size does not allow for meaningful comparison of the different sub-scores. | **No change** |
| **4.24** | *Considering the I2 values in the forest plot, re-analysis under random effects model is needed!* | We now include a re-analysis under random effects in the main text. | **Figures 3-4** |
| **4.25** | *"The upper confidence interval included no difference (i.e. zero), but the average effect favoured a reduction in IQ in babies randomised to LCPUFA". So, I believe it should be interpreted as no difference as the CI is the imp value to determine significance here.* | We have changed this to “We found no evidence that LCPUFA-supplemented infant formula improved long-term cognition among children born at term or preterm. Effect estimates were highly uncertain and consistent with potential benefit and harm” | **Discussion p. 12** |
| **4.26** | *For outcomes with substantial heterogeneity, what did the authors do to solve this heterogeneity?* | Heterogeneity could have been based on the variety of doses and sources of LCPUFA administered in the included trials. We cannot however formally assess reasons for heterogeneity due to the small number of trials. We discuss this now more explicitly. | **Discussion p.13** |
| **4.27** | *Well-written and comprehensive.* | We thank the reviewer for the positive feedback on the discussion. | **No change** |

**Response to academic editor:**

| Nr | Comment | Response | Location of change |
| --- | --- | --- | --- |
| **E.1** | *Are these figures produced by the same software mentioned in the methods, RevMan 5.3* | The figures were produced by Excel (version 16), based on the RevMan analysis results. | **p.9, Statistical methods** |
| **E.2** | *Why did you use the fixed effect model? I would advise using the random effect model instead owing to the present heterogeneity.* | Based on reviewer feedback, we have now changed this to random effects – see response to comment 4.1 for a detailed discussion of our initial reasoning for choosing fixed effects. | **Supplementary Table 1** |
| **E.3** | *Publication bias assessment is not reliable in case of fewer than 10 included studies (according to egger et al). I advise you mention it only in the supplementary files (no need to be added to the full article).* | Thank you for the opportunity to discuss how we arrived at the conclusion of potential for selective publication. We did not use the plot as the basis for our reasoning but base our concern primarily on anecdotes from trialists about the (perceived) difficulty of publishing harmful results. We have now moved the plot to the supplementary material and revised our text to state “S1 Figure illustrates this, but it should be noted that visual analysis of publication bias has limited reliability with <10 studies” | **Results p.12** |
| **E.4** | *I want to check the extracted data to repeat the analysis and compare with the reported results. Unfortunately, the raw data do not exist in the supplementary file. Please, provide them* | We added the data to the supplementary file | **Secondary analyses p.9** |
| **E.5** | *When did you use MD and when did you use SMD? Why two formats of the Effect Estimate?* | We aimed to use the mean difference (MD) when the cognitive measure was already standardised (e.g. IQ score) so as to increase interpretability, otherwise we would use the standardised mean difference (SMD). | **Outcomes and data analysis p.7** |
| **E.6** | *Did these studies report pre/post outcomes? Did you calculate the effect size based on (post - pre) or you consider the post values only for effect size calculation?* | None of the included trials used a pre/post design.  A pre / post design would not be feasible given that the outcome - cognitive ability - could only be validly measured several years after the intervention. | **No change** |
| **E.7** | *Cochrane Risk of Bias tool includes "blinding of study personnel", "blinding of outcome assessors" and "other bias" but I did not see these domains in the authors manuscript. You should include these domains in the ROB or clarify on-which basis they were omitted.* | We assume the editor is referring to Figure 6 rather than Supplementary Table 1. Figure 6 is based on the Cochrane risk of bias Excel tool (available here (<https://sites.google.com/site/riskofbiastool/welcome/rob-2-0-tool/current-version-of-rob-2>) which is available in full in Supplementary Table 1. Blinding of study personnel is included in question 2.2 (domain deviations from intended interventions), blinding of outcome assessors is included in question 4.1 (domain Measurement of the outcome). Other bias was not applicable and was not part of the tool. |  |

**Journal requirements:**

| Nr | Comment | Response |
| --- | --- | --- |
| **1.1** | *Please ensure that your manuscript meets PLOS ONE's style requirements* | Thank you, we have adjusted our manuscript accordingly. |
| **1.2** | *In your Methods section, please provide additional information about the search performed (we note that the full search string is reported as a supplementary table, but would suggest that more information is included in the main text).* | We now clarified the search strategy and added results for CENTRAL to the flow diagram. Thank you for noticing this. |
| **1.3** | *Please ensure that every statement is supported by an appropriate reference. For example, we note that the statement "No systematic review has previously evaluated the effects of LCPUFA on cognitive function in infant formula beyond early childhood (age ≥2.5 years) when cognitive tests are more accurate" is not followed by any citation corroborating the information on cognitive tests.* | We reviewed the references and added more references where appropriate. |
| **1.4** | Please include captions for your Supporting Information files at the end of your manuscript, and update any in-text citations to match accordingly. [Guidelines](http://journals.plos.org/plosone/s/supporting-information.) | Done |
| **1.5** | We note that one or more of the authors have an affiliation to the commercial funders of this research study : Health Data Research UK    1. Please provide an amended Funding Statement declaring this commercial affiliation, as well as a statement regarding the Role of Funders in your study. If the funding organization did not play a role in the study design, data collection and analysis, decision to publish, or preparation of the manuscript and only provided financial support in the form of authors' salaries and/or research materials, please review your statements relating to the author contributions, and ensure you have specifically and accurately indicated the role(s) that these authors had in your study. You can update author roles in the Author Contributions section of the online submission form. | Health Data Research UK is not a commercial funder but the national institute for health data science, an independent, non-profit organisation: https://www.sanger.ac.uk/collaboration/health-data-research-uk/ |
| **1.6** | *Please also provide an updated Competing Interests Statement declaring this commercial affiliation along with any other relevant declarations relating to employment, consultancy, patents, products in development, or marketed products, etc.* | N/A |

References:

1.	von Hippel, P.T., *The heterogeneity statistic I2 can be biased in small meta-analyses.* BMC Medical Research Methodology, 2015. **15**(1): p. 35.

2.	Huttenlocher, P.R. and A.S. Dabholkar, *Regional differences in synaptogenesis in human cerebral cortex.* J Comp Neurol, 1997. **387**(2): p. 167-78.
